# Effect of different levels of nitrogen on rhizosphere bacterial community structure in intensive monoculture of greenhouse lettuce

**DOI:** 10.1038/srep25305

**Published:** 2016-04-28

**Authors:** Jian-Gang Li, Min-Chong Shen, Jin-Feng Hou, Ling Li, Jun-Xia Wu, Yuan-Hua Dong

**Affiliations:** 1 Key Laboratory of Soil Environment and Pollution Remediation, Institute of Soil Science, Chinese Academy of Sciences, Nanjing, 210008, Jiangsu Province, P.R. China; 2 University of Chinese Academy of Sciences, Beijing, 100049, China

## Abstract

Pyrosequencing-based analyses revealed significant effects among low (N50), medium (N80), and high (N100) fertilization on community composition involving a long-term monoculture of lettuce in a greenhouse in both summer and winter. The non-fertilized control (CK) treatment was characterized by a higher relative abundance of Actinobacteria, Acidobacteria, and Chloroflexi; however, the average abundance of Firmicutes typically increased in summer, and the relative abundance of Bacteroidetes increased in winter in the N-fertilized treatments. Principle component analysis showed that the distribution of the microbial community was separated by a N gradient with N80 and N100 in the same group in the summer samples, while CK and N50 were in the same group in the winter samples, with the other N-level treatments existing independently. Redundancy analysis revealed that available N, NO_3_^−^-N, and NH_4_^+^-N, were the main environmental factors affecting the distribution of the bacterial community. Correlation analysis showed that nitrogen affected the shifts of microbial communities by strongly driving the shifts of Firmicutes, Bacteroidetes, and Proteobacteria in summer samples, and Bacteroidetes, Actinobacteria, and Acidobacteria in winter samples. The study demonstrates a novel example of rhizosphere bacterial diversity and the main factors influencing rizosphere microbial community in continuous vegetable cropping within an intensive greenhouse ecosystem.

Nitrogen (N) is the key limiting nutrient for most crops and terrestrial ecosystems. In order to reach maximal crop yields, excessive N fertilizers are often used, especially in developing countries^1^. China has been the world’s greatest consumer of N fertilizer since 1985. By 2005, China accounted for 38% of global N application, while possessing only 9% of the world’s arable land^2^. Despite new information regarding the negative effects of excessive N application, some extension staff and farmers still believe that high yield cropping systems require high amounts of N fertilizer^3^. Currently, the annual application rate of synthetic N is about 550–600 kg of N per hectare in the summer rice/winter wheat double-cropping systems in the Taihu region. On the North China Plain, the annual application rate of synthetic N has reached 550–600 kg of N per hectare in winter wheat and summer maize rotation systems^4^,^5^,^6^.

Vegetable production in China has increased over the last 20 years. During this period, some of the area devoted to cereal production was converted to intensive vegetable cultivation in plastic greenhouses. The greenhouses are able to produce three or four harvests of more profitable vegetables per year than the traditional rotation of rice/wheat (or rape)^7^. Compared with cereal production, vegetable production usually requires a greater degree of management and larger input of nutrients and irrigation, and these systems are not sustainable in the long term^8^. Nitrogen fertilization is as high as 500–1900 kg N ha^−1^yr^−1^ in China and can even be more than 3000 kg N ha^−1^yr^−1^ in some vegetable cultivation regions in North China^9^,^10^,^11^. In Shouguang, a typical greenhouse vegetable production region in Shandong Province, northern China, excessive fertilizers are commonly combined with organic manure applications to give total nitrogen (N) inputs of >1000 kg N ha^−1^ for maximum yields of vegetables^4^.

Large inputs of synthetic N fertilizer have severely disturbed soil quality^12^ and normal functioning of ecosystems^13^. Due to their ability to respond quickly to environmental changes, microorganisms are efficient bioindicators of soil biological characteristics^14^,^15^. Numerous studies have shown that changes in the size and activity of the soil microbial community are major contributors to soil degradation caused by agricultural management^16^,^17^,^18^. Soil organisms play important roles in maintaining soil health and quality and soil nutrient biogeochemical cycling, particularly in N transformation^19^,^20^,^21^. For example, both the abundance and the diversity of soil microbial communities are altered by increased N fertilization^22^,^23^,^24^,^25^ or land use intensification^26^. Recent studies have demonstrated N effects across a range of sites on both bacterial^27^,^28^, fungal communities^29^,^30^, and ammonia oxidizing bacteria^31^. Giagnoni *et al*. also found availability of different N forms was a dominant factor regulating activity and composition of the rhizosphere microbial communities. In addition, N supply rate could change the composition of bacterial, fungal and AOB communities^32^. However, the effects of excessive N inputs in vegetable production systems on the soil microbial community and their attendant functions have not been fully elucidated. Moreover, little is known about the influences of N management regimes on belowground soil microbial community properties in intensive monocultured greenhouse vegetable systems.

In our study, we established a greenhouse in which lettuce plants were grown continuously for 4 years with six crops per year. The bacterial communities inhabiting the rhizospheric soil were detected using pyrosequencing at different levels of N fertilization. The aims of this work were to: 1) investigate the effect of the N fertilizer application on microbial composition and structure and their changes in different seasons, and 2) explore key bacteria that are significantly correlated with N fertilization application in intensively cultivated greenhouse systems. We hypothesized that the bacterial diversity was different from other ecosystems due to the particular management present in greenhouse vegetable cultivation.

## Results

### Overall Diversity of Bacterial Communities

Total microbial communities were characterized by high-throughput pyrosequencing of rhizosphere soil from summer and winter samples in triplicate. Across all 24 samples (12 summer and 12 winter samples), a total of 1,213,034 high-quality sequences (670,908 sequences for summer and 542,126 for winter samples) were obtained after the low quality reads were removed. There were an average of 52,168, 70,601, 39,304 and 61,562 effective sequence reads for CK, N50, N80, and N100 in summer samples, and 42,258, 46,590, 41,373, and 50,485 effective sequence reads in winter samples for CK, N50, N80, and N100, respectively. The effective reads in the summer were more than the winter samples except for the N80 treatment (Table 1). The highest operation taxonomic unit (OTU) number was observed in no fertilizer control (CK) treatment in the winter sample, (2,305 at a distance cutoff level of 3%) (Table 1), while the lowest number of OTUs was obtained for the N50 treatment of the summer sample. There were no significant changes in OTUs across the N gradient in winter samples (P > 0.05). CK in winter showed the highest bacterial diversity among the eight treatments, as shown by the Shannon diversity and Chao 1 index, which are representative of bacterial phylotype richness and evenness levels (Table 1). The richness of CK treatments in either summer or winter samples was higher than other fertilized treatments (Table 1). In the summer samples, there was a significant decrease in Shannon diversity and the Chao 1 index in response to nitrogen addition between N50 and N80 (Duncan analysis, p < 0.05). However, in the winter samples, the N addition treatment did not significantly change Shannon diversity (Table 1).

**Figure 1:**
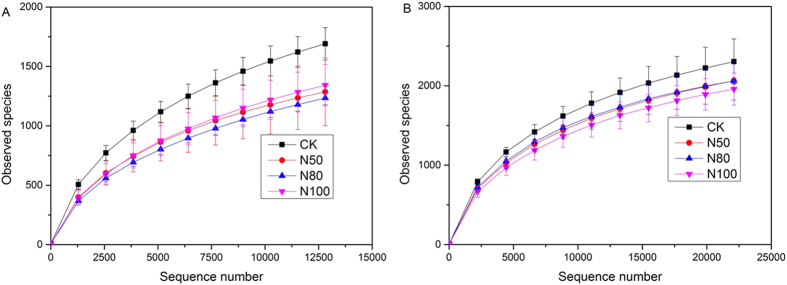
Rarefaction curves of observed species of 16S rRNA genes from the summer. (**A**) and winter (**B**) soil samples based on 97% similarity. The error bars of observed species indicate standard deviations of three replicates. 100N: normal application rate of N, P_2_O_5_, and K_2_O was 185, 46, and 185 kg ha^−1^, respectively; 80N: 0.8 times N as in the 100N; 50N: 0.5 times N as in the 100N; CK: no N applied.

Rarefaction curves were used to estimate and compare bacterial diversity and richness among different treatments in summer and winter samples at a distance cutoff level of 3% as shown in Fig. 1. All amplified rarefaction curves increased rapidly from 0 to 1000 sequences, indicating that sequence-derived diversity and richness in this study were sufficient to characterize the species in each sample. The rarefaction curves showed that the bacterial richness were higher in the winter sample than those of summer samples. Compared to CK, treatments of N50, N80, and N100 consistently decreased the observed OTU richness (observed species) (Fig. 1).

### Bacterial Community Composition in the rhizosphere

**Figure 2:**
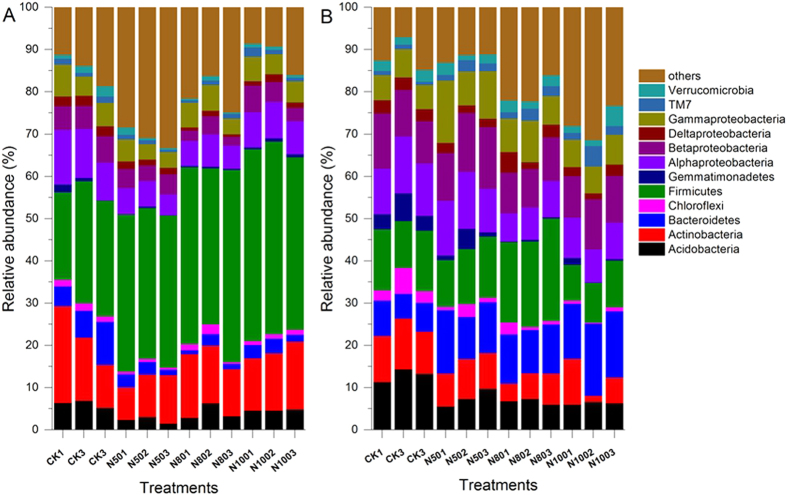
Relative abundances of the dominant bacterial phylotypes in the summer. (**A**) and winter (**B**) soil samples. Relative abundances are based on the proportional frequencies of DNA sequences that could be classified at the phylum level. 100N: normal application rate of N, P_2_O_5_, and K_2_O was 185, 46, and 185 kg ha^−1^, respectively; 80N: 0.8 times N as in the 100N; 50N: 0.5 times N as in the 100N; CK: no N applied.

The effective bacterial sequences in the 24 samples were all assigned to corresponding taxonomies using BLAST combined with MEGAN. The dominant phyla across all the samples were Proteobacteria, Firmicutes, Actinobacteria, Acidobacteria, and Bacteroidetes, accounting for more than 65% of the bacterial sequences from each of the soils (Fig. 2, Tables S1 and S2). In addition, Verrucomicrobia, Gemmatimonadetes, Chloroflexi, and TM7 were present in most rhizosphere soils but at relatively low abundances, and 9 other more rare phyla were also identified (Tables S1 and S2).

In the summer soil samples, Firmicutes was on average the dominant phylum, accounting for 20.79–45.60% of the total 16S rRNA gene reads sequences, and the relative abundance of the Proteobacteria (11.95–28.71%) was second only to the Firmicutes (Fig. 2A, Table S1). However, the winter samples showed an opposite trend, the most dominant phylum was the Proteobacteria, amounting to 28.26–41.83%; and the second was Firmicutes, with the relative abundance of 8.46–24.26% (Fig. 2B, Table S2). The relative abundance of Acidobacteria and Bacteroidetes were lower in summer soil samples than in winter soil samples, but Actinobacteria abundance showed an opposite trend (Fig. 2, Tables S1 and S2).

The distribution of each phylum among the different treatments was evaluated. CK treatment was characterized by a higher average of abundance of Actinobacteria, Acidobacteria, and Chloroflexi (Fig. 2). We also compared the relative abundance in the same treatments of different seasons; the results showed that the dominant phyla were similar in summer and winter, although with different amounts (Fig.S1). In order to further to examine differences in bacterial community composition between summer and winter samples, a multiple-response permutation procedure (MRPP) was employed to compare the difference in the same treatment (with the same N fertilizer application) of summer and winter samples, but there was no significance in the same N level treatment from both seasons (P > 0.05). These results showed that the rhizosphere bacterial composition varied as the season changed and maintained similar trends between N-fertilized and non-N-fertilized CK.

### Differences in Bacterial Communities among Fertilizations

**Figure 3:**
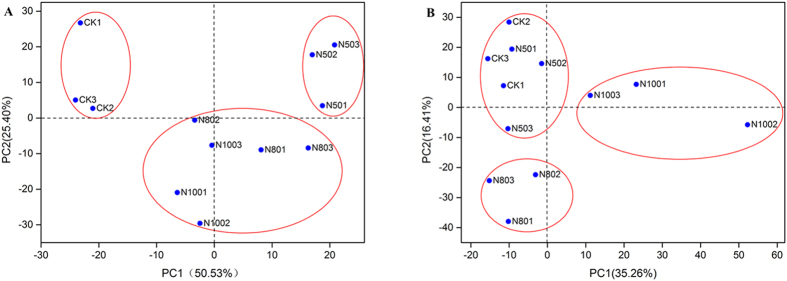
Principal component analyses (PCA) of shifts in soil under different levels of N fertilization for in the summer. (**A**) and winter (**B**) soil samples. 100N: normal application rate of N, P_2_O_5_, and K_2_O was 185, 46, and 185 kg ha^−1^, respectively; 80N: 0.8 times N as in the 100N; 50N: 0.5 times N as in the 100N; CK: no N applied.

Principal component analyses (PCA) demonstrated clear distinctions in the bacterial communities under different fertilization management regimes in summer and winter soil samples (Fig. 3). In the summer samples, the first principal component (50.53% of contribution rate), which explains the majority of variations in the data, clearly separated all fertilized and unfertilized CK soils. The second principal component axis (25.40% of contribution rate) differentiated bacterial communities in controls and N50s from those in N80s and N100s, which suggests strong variations in bacterial community structure between N-deficient and N-rich fertilization soils (Fig. 3A). In the winter soil samples, both the first and second principal components mainly separated the community composition by differences in the application rates of N fertilizer which was the only difference among the treatments (Fig. 3B). In the summer samples, N-rich treatments (N80s and N100s) harbored similar bacterial community composition, while in the winter samples, the N-deficient treatments (CKs and N50s) formed a separated group (Fig. 3), and the rhizosphere microbial communities were well separated from the medium and high N treatments. These results suggest that the change in seasons also brings changes in the bacterial community structure.

### Effect of Environmental Factors on Bacterial Community at Different Sampling Season

**Figure 4:**
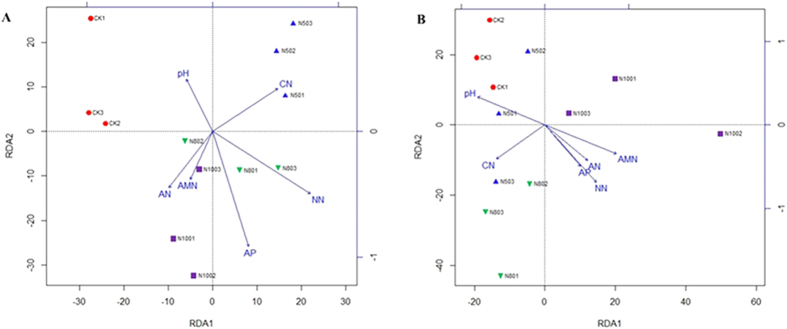
Redundancy analysis (RDA) of the bacterial communities with environmental variables in different N-level fertilized soils for summer. (**A**) and winter (**B**) soil samples. 100N: normal application rate of N, P_2_O_5_, and K_2_O was 185, 46, and 185 kg ha^−1^, respectively; 80N: 0.8 times N a as in the 100N; 50N: 0.5 times N as in the 100N; CK: no N applied. C/N: C/N ratio; pH: soil pH value; AP: available P; AN: available N; NN: NO_3_^−^-N; AMN: NH_4_^+^-N.

Redundancy analysis (RDA) was used to reveal what environmental factors shifted bacterial assemblages in rhizosphere. Environmental variables included pH value, C/N ratio (CN), available N (AN) and P (AP), nitrate N (NN), and ammonium N (AMN). Statistical analysis showed that environmental variables were different between control and long-term N-fertilized treatments (Fig. 4). In the summer samples, the nitrate N (NN) was the strongest factor (P = 0.002) that was correlated with the distribution of phyla. Available P (AP) also showed marginally significant correlations with community composition (P = 0.039), while the other factors were all non-significant (Fig. 4A). The relationship between environmental variables and microbial composition revealed that controls had higher pH value, while N50s were more related to C/N ratio. The higher contents of AN, AP, NN, and AMN were observed for N80 and N100 treatments, indicating the bacterial distribution in the rhizosphere was correlated with the application rate of N fertilizer (Fig. 4A). In the winter samples, the effect of environmental factors on the microbial community composition resulted in minor changes compared with summer samples. Available N (AN) and ammonium N (AMN) showed marginally significant correlations with community composition (P = 0.018 and P = 0.028, respectively) (Fig. 4B). Besides the CK treatment, pH also influenced the N50 treatment. The bacterial species in the N80s were positively affected by C/N ratio, but negative correlated with N100s (Fig. 4B). All of the results for both summer and winter samples indicated that the N fertilizer content shifted the distribution of the bacterial community, although the main factor influencing the changes was different.

### Relationship between N Gradient and Dominant Phyla

**Figure 5:**
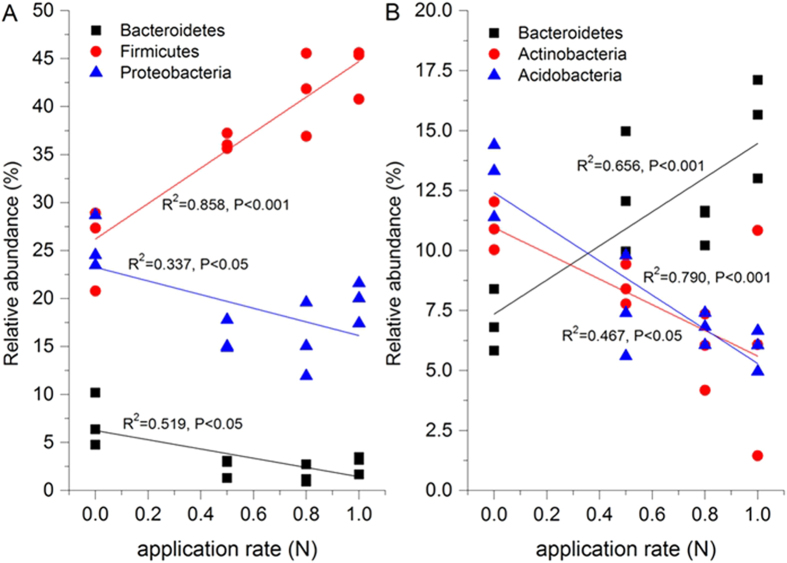
The relationships between relative abundances of dominant bacterial groups and N gradients. Linear regressions were used to test the correlation between the relative abundances of the taxa and N application. P < 0.05. 100N: normal application rate of N, P_2_O_5_, and K_2_O was 185, 46, and 185 kg ha^−1^, respectively; 80N: 0.8 times N as in the 100N; 50N: 0.5 times N as in the 100N; CK: no N applied.

Linear regression analysis was employed to further examine the effect of the N application gradient on the relative abundances of the dominant bacterial phyla (Actinobacteria, Bacteroidetes, Acidobacteria, proteobacteria, and Firmicutes) in both summer and winter samples (Fig. 5). The N application rate was significantly positively correlated with Firmicutes (R^2^ = 0.858, P < 0.001), while it was also strongly negatively correlated with Bacteroidetes (R^2^ = 0.519, P < 0.05) and proteobacteria (R^2^ = 0.337, P < 0.05) in summer samples (Fig. 5A). In the winter samples, we observed a strong positive correlation between N gradient and Bacteroidetes (R^2^ = 0.656, P < 0.001), but a negative correlation with Actinobacteria (R^2^ = 0.467, P < 0.05) and Acidobacteria (R^2^ = 0.790, P < 0.001) (Fig. 5B).

## Discussion

Soil microorganisms are vital to the environment due to their role in cycling mineral compounds, decomposing organic materials, and various soil biophysical processes^33^,^34^. In turn, soil microbial populations exhibit natural seasonal dynamics for their biodiversity and functions are influenced by various factors including vegetation, soil nutrients, and agricultural management^33^,^35^. In China, a large amount of traditional croplands have been recently transformed into high N input greenhouse vegetable production systems. Recently, significant effects have been observed from current soil management practices on soil quality and sustainability in the polytunnel greenhouse vegetable systems^7^,^36^. Intense management and excessive N application in the greenhouse fields inevitably creates microbial communities distinct from those found on traditional farming land or other ecosystems.

In the present study, a long-term fertilization experiment was conducted in greenhouse fields, in which the lettuce was mono-cultured six times each year with the same fertilizer regimes for four years. The summer and winter soil samples of the fourth year were collected separately. We observed that the soils from the CK treatment had higher levels of bacterial taxonomic diversity than the N application treatments exhibited by the Chao1 index, Shannon diversity, and rarefaction curve (Table 1; Fig. 1). Reduction in diversity in fertilized plots has also been observed in the arctic tundra^27^,^37^, suggesting that nitrogen enrichment may reduce niche space and therefore community diversity. In contrast, a study in another grassland ecosystem (Cedar Creek) observed no reduction in diversity in response to nitrogen enrichment, indicating that species richness responses to nitrogen enrichment may be site or soil specific^38^. More comprehensive cross-site studies are required to test this hypothesis.

Soil bacterial community composition is altered in response to N addition in both seasons. In the summer, Firmutes and many of the dominant Actinobacteria and Acidobacteria groups increased in relative abundance across the N gradient (Table S1, Fig. 2A); while in the winter, the relative abundance of Actinobacteria and Acidobacteria decreased, but Bacteroidetes increased with the N addition (Table S1, Fig. 2B). Similar taxon shifts have been found in other studies examining bacterial community responses to N addition. For example, Campbell *et al*.^27^ noted that Acidobacteria were relatively less abundant in fertilized samples, a pattern also observed by Wessen *et al*.^28^. A study of multiple terrestrial ecosystems showed that nitrogen enrichment significantly shifted bacterial communities^39^, suggesting that this may be a more general effect of N enrichment. One interpretation of this phenomenon may be that there are threshold responses in microbial community changes to enhanced soil nitrogen availability, and the changes associated with high level N application elicit the responses and accordingly drive the shifts in the bacterial community^37^.

Soil microorganisms play important roles in soil nutrient biogeochemical cycling, particularly in N transformation^20^. Compared with other cultivation systems, polytunnel greenhouse vegetable land in China is characterized with year-round cultivation and excessive N fertilizer application, which results in significant changes in soil chemical properties with accumulation of soil N, P, and K, as well as lower N use efficiency, and acidification as soil pH sharply decreased^26^. In our study, results showed that environmental variables were different between control and long-term fertilization treatments (Fig. 4). In the summer samples, controls had higher pH values, indicating pH may also be an important factor controlling soil microbial communities^40^. While N50s were more related to C/N ratio, the higher contents of available N, NO_3_^−^-N, and NH_4_^+^-N were observed for N80 and N100 treatments (Fig. 4A). In the winter samples, environmental factors created changes in microbial community composition compared with summer samples (Fig. 4B). The bacterial species in the N80s were affected by C/N ratio, while in the N100s, they were negatively influenced by C/N ratio (Fig. 4B). These results for both summer and winter samples further confirmed that the distribution of the bacterial community mainly induced by N nutrition in the soil originated from N fertilizer. Shen *et al*.^26^ also found that both the abundance and the diversity of soil microbial communities were altered by increased N fertilization or land use intensification.

The correlation between the N gradient and dominant bacterial phyla was also investigated to examine the key bacteria that respond to N addition (Fig. 5). Interestingly, we found that the N level exhibited a distinct negative correlation with Bacteroidetes in the summer (R^2^ = 0.519, P < 0.05, Fig. 5A), but demonstrated a strong positive correlation between N gradient and Bacteroidetes in the winter (R^2^ = 0.656, P < 0.001, Fig. 5B). This may be due to the distinct differences in climatic conditions, such as temperature and humidity, which influence nutrient cycling and the interaction between plants and microorganisms in the soil ecosystem.

Season was a factor influence microbial community shift in the same plots, however, some phyla in the same N level between summer and winter had the similar shift trend, in which the bacterial communities receiving the highest levels of N addition were significantly different from the communities receiving intermediate levels or no added N (Fig. S1), and there was no significant difference between them when analyzed by the MRPP method (P > 0.05). The bacterial diversity, community composition, and structure variation in winter samples showed distinct characteristics compared with summer samples. These possible mechanisms need further investigation to explain soil microbial responses to N amendments.

The data presented here showed that N addition can significantly alter bacterial community diversity and structure, and successfully identified key bacteria phyla that respond to excessive N input in intensively cultivated greenhouse soils with a long-term monoculture of lettuce. However, a more detailed study would be required to explain the mechanisms in this process.

## Methods

### Site and Experimental Design

The experimental site was in a demonstration garden in Wangting, Jiangsu Province, China (36°26′N, 120°27′E). Average annual rainfall is 1100 mm with mean air temperatures of 2.5 and 28 °C in January and July, respectively. Soil was characterized by a pH of 5.58, and the contents of organic matter, available N and available P were 28.05, 147.06, and 9.82 mg kg^−1^, respectively, at the beginning of the experiment. The vegetable cultivar tested was lettuce (*Lactuca sativa* L. var. ramosa Hort.), which was continuously sown from the beginning of 2010 to the end of 2013 with harvests six times each year (The last soil sample was collected in February 2014). Twenty-four crops in total were harvested from the beginning of 2010 to the end of 2013. Each time, the normal application rate of N, P_2_O_5_, and K_2_O was 185, 46, and 185 kg ha^−1^, respectively (100N). The treatments consisted of four fertilizer regimes in a completely random plot design with three replicates including: (1) 100N (applied as urea, superphosphate, and potassium sulfate, respectively); (2) 80N (0.8 times N and the same rate of PK application as in the 100N treatment); (3) 50N (0.5 times N and the same rate of PK applied as in the 100N treatment); (4) CK (no N applied), Each plot has received the same fertilizer management since 2010. The plot size was 1.8 × 2.4 m^2^. In each plot, 96 plants of the same variety were planted after each harvest. The fertilizer was a one-time application.

### Soil Sample Collection

The soil samples were separately collected twice at the time of the lettuce harvest in August 2013 (summer) and February 2014 (winter). The rhizospheric soil samples were collected as described by Li *et al*.^41^. Each soil sample was stored at 4 °C until it was brought to the lab for immediate processing. Soil samples were homogenized by filtering through a 2-mm sieve to remove aboveground plant materials, roots, and stones for downstream processing, including microbial genomic DNA extraction. For soil chemical parameter analysis, a soil auger was inserted to 20 cm soil depth in order to collect soil samples from each plot, and five soil samples were randomly collected from each plot. Soil samples were air dried at room temperature and sieved to pass a 2-mm mesh.

### Soil Characteristics Analysis

All three replicates were used for soil chemistry analysis. Soil pH was determined with a glass electrode using a soil-to-water ratio of 1:2.5. Soil organic C (SOC) and total N (TN) were determined by dichromate oxidation^42^ and Kjeldahl digestion^43^. Total P was determined by the Vanado-Molybdate phosphoric yellow colorimetric procedure^44^. Available P was analyzed by the method of Olsen *et al*.^45^. Available N was estimated by the alkaline permanganate method suggested by Subbiah and Asija^46^. The concentrations of NH_4_^+^-N and NO_3_^−^-N in the soil samples were analyzed as described by Turner and Romero^47^.

### Soil DNA Extraction

Genomic DNA was extracted from 0.5 g of each soil sample using a FastDNA SPIN Kit for soil (MP Biomedicals, Santa Ana, CA, USA) according to the manufacturer’s instructions. The extracted soil DNA was dissolved in 70 μL of TE buffer, quantified by spectrophotometry, and stored at −20 °C for further analysis. To isolate genomic DNA from endophytic microbial communities, tomato roots were washed under running water, and surface sterilized by immersion in 70% ethanol (60 s) followed by treatment in 0.1% (w/v) mercuric chloride for 10 min. Surface sterilization of the plant material was checked by rolling the sterilized plant material on nutrient agar plates, which were then incubated for up to 7 days at 28 °C.

### PCR Amplification and Pyrosequencing

The V4 region of the 16S rRNA genes were amplified using the universal primers set 515F (5′-GTGCCAGCMGCCGCGGTAA-3′) and 806R (5′-GGACTACVSGGGTATCTAAT-3′) with the barcode^48^. All PCR reactions were carried out in 30 μL reactions with 15 μL of Phusion® High-Fidelity PCR Master Mix (New England Biolabs), 0.2 μM of forward and reverse primers, and about 10 ng template DNA. Thermal cycling consisted of initial denaturation at 98 °C for 1 min, followed by 30 cycles of denaturation at 98 °C for 10 s, annealing at 50 °C for 30 s, elongation at 72 °C for 60 s, and, finally, 72 °C for 5 min. The triplicate PCR amplicons were mixed with the same volume of 1× loading buffer (containing SYBR green) and subjected to electrophoresis on 2% agarose gels for detection. Samples with a bright main band of between 400–450 bp were chosen for further experiments. Then, mixture PCR products were purified with a GeneJET Gel Extraction Kit (Thermo Scientific).

Sequencing libraries were generated using NEB Next® Ultra™ DNA Library Prep Kit for Illumina (NEB, USA) following the manufacturer’s recommendations (index codes were added). The library quality was assessed on the Qubit@ 2.0 Fluorometer (Thermo Scientific) and an Agilent Bioanalyzer 2100 system. Finally, the library was sequenced on an Illumina MiSeq platform and 250 bp/300 bp paired-end reads were generated. Sequence analysis was performed by the UPARSE software package using the UPARSE-OTU and UPARSE-OTUref algorithms^49^. In-house Perl scripts were used to analyze alpha (within samples) and beta (among samples) diversity. Sequences with ≥97% similarity were assigned to the same OTUs. We chose representative sequences for each OTU and used the RDP classifier to annotate taxonomic information for each representative sequence^50^. In order to compute alpha diversity, we rarified the OTU table and calculated three metrics: Chao1 estimates of speciesrichness; observed species estimates of the amount of unique OTUs found in each sample, and the Shannon index. Rarefaction curves were generated based on these three metrics.

### Data Analysis

Cluster analysis was preceded by principal component analysis (PCA) to evaluate the degree of similarity between microbial communities associated with different samples. Redundancy analysis (RDA) was chosen to examine the relationship between the bacterial community and environmental factors. Multiple-response permutation procedure (MRPP) was performed with Bray-Curtis distances to examine the difference between the same treatment in the summer and winter. The PCA, RDA, and MRPP were calculated used assigned OTUs using R with “vegan” package^51^, which were performed at the phylum level. The significance of the effects of N level effects on microbial pyrosequencing reads and diversity was analyzed by analysis of variance (ANOVA) for repeated measures with the statistical software package SPSS 20.0 (SPSS Inc.). Statistical analyses were run in Origin 9.0.

## Additional Information

**How to cite this article**: Li, J.-G. *et al*. Effect of different levels of nitrogen on rhizosphere bacterial community structure in intensive monoculture of greenhouse lettuce. *Sci. Rep*. **6**, 25305; doi: 10.1038/srep25305 (2016).

## Supplementary Material

Supplementary Information

## Figures and Tables

**Table 1:** Summary of pyrosequence reads (Mean ± SE, n = 3).

Samples	Effective Reads		No. of OTUs*		Shannon		Chao1	
	Summer	Winter	Summer	Winter	Summer	Winter	Summer	Winter
CK	52168 ± 3277ab	42258 ± 5938a	1753 ± 34c	2305 ± 179a	8.68 ± 0.19c	9.15 ± 0.03a	2473 ± 32b	3033 ± 424a
N50	70601 ± 9344b	46590 ± 1751a	1271 ± 35a	2063 ± 24a	7.22 ± 0.08a	8.76 ± 0.04a	1892 ± 30a	2664 ± 42a
N80	39304 ± 3664a	41373 ± 7851a	1333 ± 78ab	2061 ± 152a	7.52 ± 0.29ab	8.74 ± 0.15a	1941 ± 93a	2577 ± 260a
N100	61562 ± 4887b	50485 ± 457a	1497 ± 37b	1959 ± 129a	7.82 ± 0.04b	8.57 ± 0.32a	2269 ± 84b	2522 ± 157a

^*^Operational taxonomic units (OTUs) were defined at 97% sequence identity. 100N: normal application rate of N, P_2_O_5_, and K_2_O was 185, 46, and 185 kg ha^−1^, respectively; 80N: 0.8 times N a as in the 100N; 50N: 0.5 times N as in the 100N; CK: no N applied. Means followed by the same letter within a column are not significantly different as determined by the LSD test (P = 0.05).
