# Supplementary material for: Effect of different levels of nitrogen on rhizosphere bacterial community structure in intensive monoculture of greenhouse lettuce
Source: Sci Rep. 2016 Apr 28;6:25305. doi: 10.1038/srep25305 (PMC4848521; doi:10.1038/srep25305)
Supplement: Supplementary Information [file srep25305-s1.pdf]

Title page

Title: Effect of different levels of nitrogen on rhizosphere bacterial community structure in intensive monoculture of greenhouse lettuce

Author: Jian-Gang Li[1], Min-Chong Shen[1,2], Jin-Feng Hou[1,2], Ling Li[1,2], Jun-Xia Wu[1], Yuan-Hua Dong[1]*

Affiliation:

[1] Key Laboratory of Soil Environment and Pollution Remediation

Institute of Soil Science, Chinese Academy of Sciences

Nanjing, 210008, Jiangsu Province, P.R. China

[2] University of Chinese Academy of Sciences

Beijing, 100049, China

*** Corresponding author**

E-mail: yhdong@issas.ac.cn

Tel: +86-25-8688-1317

Fax: +86-25-8688-1317

Table S1 Relative abundance of the identified main phylotypes in summer samples. Note: 100N: normal application rate of N, P_2O_5, and K_2O was 185, 46, and 185 kg ha^{-1}, respectively; 80N: 0.8 times N a as in the 100N; 50N: 0.5 times N as in the 100N; CK: no N applied.

Taxon	CK1	CK2	CK3	N501	N502	N503	N801	N802	N803	N1001	N1002	N1003
Acidobacteria	0.0647	0.0697	0.0530	0.0245	0.0315	0.0155	0.0293	0.0635	0.0327	0.0464	0.0461	0.0492
Actinobacteria	0.2284	0.1493	0.1017	0.0773	0.1003	0.1144	0.1507	0.1364	0.1120	0.1236	0.1357	0.1604
Bacteroidetes	0.0476	0.0638	0.1019	0.0311	0.0296	0.0129	0.0092	0.0273	0.0118	0.0318	0.0347	0.0166
Chloroflexi	0.0146	0.0176	0.0130	0.0053	0.0076	0.0051	0.0144	0.0233	0.0041	0.0095	0.0105	0.0119
Firmicutes	0.2079	0.2890	0.2736	0.3723	0.3566	0.3601	0.4186	0.3690	0.4556	0.4536	0.4560	0.4078
Gemmatimonadetes	0.0183	0.0075	0.0009	0.0005	0.0041	0.0009	0.0034	0.0040	0.0025	0.0048	0.0076	0.0071
Proteobacteria	0.2871	0.2454	0.2348	0.1780	0.1486	0.1502	0.1504	0.1959	0.1195	0.2162	0.2003	0.1743
TM7	0.0138	0.0089	0.0151	0.0113	0.0091	0.0055	0.0059	0.0102	0.0115	0.0217	0.0110	0.0086
Verrucomicrobia	0.0092	0.0161	0.0247	0.0168	0.0052	0.0039	0.0037	0.0105	0.0028	0.0089	0.0075	0.0059
Armatimonadetes	0.0030	0.0021	0.0023	0.0004	0.0005	0.0003	0.0006	0.0023	0.0005	0.0006	0.0012	0.0006
Chlamydiae	0.0032	0.0010	0.0023	0.0008	0.0005	0.0008	0.0005	0.0018	0.0007	0.0019	0.0010	0.0012
Chlorobi	0.0009	0.0002	0.0002	0.0002	0.0002	0.0001	0.0000	0.0001	0.0004	0.0005	0.0004	0.0001
Cyanobacteria	0.0093	0.0162	0.1080	0.0155	0.0119	0.0164	0.0064	0.0076	0.0098	0.0063	0.0108	0.0137
Elusimicrobia	0.0003	0.0009	0.0012	0.0001	0.0002	0.0002	0.0000	0.0002	0.0001	0.0002	0.0005	0.0001
Nitrospirae	0.0045	0.0059	0.0024	0.0007	0.0022	0.0019	0.0018	0.0023	0.0019	0.0032	0.0023	0.0024
Planctomycetes	0.0053	0.0055	0.0077	0.0079	0.0052	0.0051	0.0052	0.0102	0.0023	0.0028	0.0052	0.0059
WPS-2	0.0020	0.0020	0.0016	0.0034	0.0038	0.0026	0.0035	0.0030	0.0040	0.0035	0.0028	0.0037
others	0.0798	0.0990	0.0556	0.2540	0.2831	0.3042	0.1964	0.1324	0.2279	0.0645	0.0663	0.1306

Table S2 Relative abundance of the identified main phylotypes in cwinter samples. Note: 100N: normal application rate of N, P_2O_5, and K_2O was 185, 46, and 185 kg ha^{-1}, respectively; 80N: 0.8 times N a as in the 100N; 50N: 0.5 times N as in the 100N; CK: no N applied.

Taxon	CK1	CK2	CK3	N501	N502	N503	N801	N802	N803	N1001	N1002	N1003
Acidobacteria	0.1140	0.1440	0.1332	0.0560	0.0739	0.0980	0.0683	0.0741	0.0606	0.0606	0.0666	0.0496
Actinobacteria	0.1090	0.1203	0.1004	0.0778	0.0944	0.0841	0.0418	0.0605	0.0736	0.1084	0.0145	0.0608
Bacteroidetes	0.0840	0.0583	0.0681	0.1498	0.0997	0.1206	0.1166	0.1021	0.1158	0.1301	0.1711	0.1566
Chloroflexi	0.0241	0.0615	0.0269	0.0084	0.0303	0.0109	0.0281	0.0078	0.0083	0.0076	0.0024	0.0099
Firmicutes	0.1445	0.1104	0.1436	0.1107	0.1308	0.1444	0.1901	0.2019	0.2426	0.0846	0.0941	0.1102
Gemmatimonadetes	0.0356	0.0659	0.0352	0.0107	0.0473	0.0101	0.0024	0.0048	0.0036	0.0166	0.0011	0.0038
Proteobacteria	0.3382	0.3578	0.3134	0.4183	0.3967	0.3851	0.2936	0.3146	0.2915	0.3101	0.2826	0.3069
TM7	0.0098	0.0101	0.0079	0.0130	0.0263	0.0186	0.0154	0.0284	0.0231	0.0162	0.0485	0.0204
Verrucomicrobia	0.0246	0.0178	0.0278	0.0292	0.0120	0.0212	0.0273	0.0169	0.0254	0.0160	0.0142	0.0475
Armatimonadetes	0.0026	0.0033	0.0035	0.0018	0.0016	0.0025	0.0012	0.0014	0.0021	0.0024	0.0002	0.0015
Chlamydiae	0.0017	0.0012	0.0019	0.0015	0.0009	0.0023	0.0020	0.0028	0.0029	0.0015	0.0039	0.0013
Chlorobi	0.0010	0.0010	0.0012	0.0009	0.0007	0.0010	0.0025	0.0013	0.0010	0.0009	0.0005	0.0005
Cyanobacteria	0.0161	0.0131	0.0620	0.0110	0.0289	0.0783	0.0556	0.0746	0.0386	0.1382	0.2495	0.1492
Elusimicrobia	0.0015	0.0011	0.0011	0.0004	0.0003	0.0010	0.0010	0.0005	0.0017	0.0009	0.0002	0.0009
Fibrobacteres	0.0006	0.0005	0.0010	0.0005	0.0007	0.0004	0.0005	0.0003	0.0004	0.0009	0.0022	0.0008
Nitrospirae	0.0083	0.0057	0.0071	0.0038	0.0032	0.0033	0.0125	0.0011	0.0033	0.0039	0.0008	0.0020
OD1	0.0013	0.0009	0.0020	0.0021	0.0014	0.0018	0.0010	0.0024	0.0029	0.0022	0.0025	0.0023
Planctomycetes	0.0055	0.0089	0.0087	0.0059	0.0130	0.0037	0.0038	0.0105	0.0078	0.0067	0.0019	0.0084
WPS-2	0.0009	0.0017	0.0011	0.0013	0.0053	0.0018	0.0017	0.0049	0.0017	0.0018	0.0011	0.0012
others	0.0769	0.0164	0.0540	0.0966	0.0329	0.0109	0.1344	0.0894	0.0934	0.0905	0.0422	0.0663

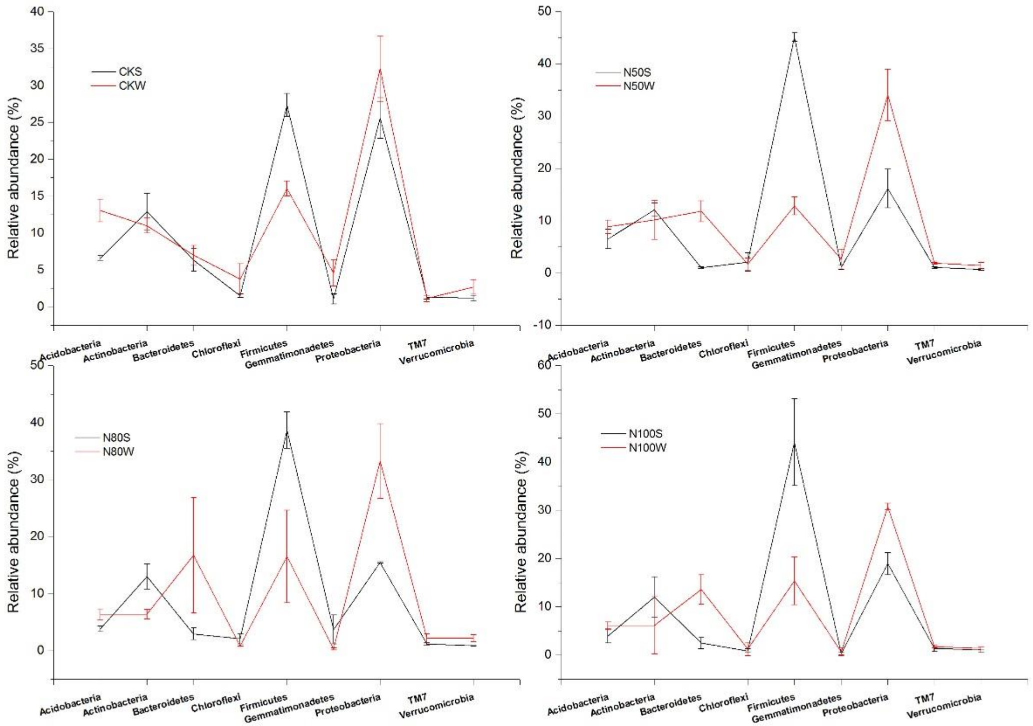

Figure S1. The relative abundance of dominant phyla in the same treatments of summer (S) and winter (W) samples. 100N: normal application rate of N, P_2O_5, and K_2O was 185, 46, and 185 kg ha^{-1}, respectively; 80N: 0.8 times N as in the 100N; 50N: 0.5 times N as in the 100N; CK: no N applied.
